# Impact of Socioeconomic Deprivation on Care Quality and Surgical Outcomes for Early-Stage Non-Small Cell Lung Cancer in United States Veterans

**DOI:** 10.3390/cancers16223788

**Published:** 2024-11-11

**Authors:** Steven Tohmasi, Daniel B. Eaton, Brendan T. Heiden, Nikki E. Rossetti, Ana A. Baumann, Theodore S. Thomas, Martin W. Schoen, Su-Hsin Chang, Nahom Seyoum, Yan Yan, Mayank R. Patel, Whitney S. Brandt, Bryan F. Meyers, Benjamin D. Kozower, Varun Puri

**Affiliations:** 1 Division of Cardiothoracic Surgery, Department of Surgery, Washington University School of Medicine, St. Louis, MO 63110, USA; 2 Veterans Affairs St. Louis Health Care System, St. Louis, MO 63110, USA; 3 Division of Public Health Sciences, Department of Surgery, Washington University School of Medicine, St. Louis, MO 63110, USA; 4 Division of Oncology, Department of Medicine, Washington University School of Medicine, St. Louis, MO 63110, USA; 5 Division of Hematology and Medical Oncology, Department of Internal Medicine, Saint Louis University School of Medicine, St. Louis, MO 63104, USA

**Keywords:** socioeconomic deprivation, area deprivation index, lung cancer, non-small cell lung cancer, lung cancer surgery, cancer disparities, cancer outcomes, preoperative care, readmission, Veteran Affairs, Veterans Health Administration, veterans

## Abstract

**Background**: Socioeconomic deprivation has been associated with higher lung cancer risk and mortality in non-Veteran populations. However, the impact of socioeconomic deprivation on outcomes for non-small cell lung cancer (NSCLC) in an integrated and equal-access healthcare system, such as the Veterans Health Administration (VHA), remains unclear. Hence, we investigated the impact of area-level socioeconomic deprivation on access to care and postoperative outcomes for early-stage NSCLC in United States Veterans. **Methods**: We conducted a retrospective cohort study of patients with clinical stage I NSCLC receiving surgical treatment in the VHA between 1 October 2006 and 30 September 2016. A total of 9704 Veterans were included in the study and assigned an area deprivation index (ADI) score, a measure of socioeconomic deprivation incorporating multiple poverty, education, housing, and employment indicators. We used multivariable analyses to evaluate the relationship between ADI and postoperative outcomes as well as adherence to guideline-concordant care quality measures (QMs) for stage I NSCLC in the preoperative (positron emission tomography [PET] imaging, appropriate smoking management, pulmonary function testing [PFT], and timely surgery [≤12 weeks after diagnosis]) and postoperative periods (appropriate surveillance imaging, smoking management, and oncology referral). **Results**: Compared to Veterans with low socioeconomic deprivation (ADI ≤ 50), those residing in areas with high socioeconomic deprivation (ADI > 75) were less likely to have timely surgery (multivariable-adjusted odds ratio [aOR] 0.832, 95% confidence interval [CI] 0.732–0.945) and receive PET imaging (aOR 0.592, 95% CI 0.502–0.698) and PFT (aOR 0.816, 95% CI 0.694–0.959) prior to surgery. In the postoperative period, Veterans with high socioeconomic deprivation had an increased risk of 30-day readmission (aOR 1.380, 95% CI 1.103–1.726) and decreased odds of meeting all postoperative care QMs (aOR 0.856, 95% CI 0.750–0.978) compared to those with low socioeconomic deprivation. There was no association between ADI and overall survival (adjusted hazard ratio [aHR] 0.984, 95% CI 0.911–1.062) or cumulative incidence of cancer recurrence (aHR 1.047, 95% CI 0.930–1.179). **Conclusions**: Our results suggest that Veterans with high socioeconomic deprivation have suboptimal adherence to care QMs for stage I NSCLC yet do not have inferior long-term outcomes after curative-intent resection. Collectively, these findings demonstrate the efficacy of an integrated, equal-access healthcare system in mitigating disparities in lung cancer survival that are frequently present in other populations. Future VHA policies should continue to target increasing adherence to QMs and reducing postoperative readmission for socioeconomically disadvantaged Veterans with early-stage NSCLC.

## Simple Summary

Disparities in outcomes for non-small cell lung cancer (NSCLC) may result from socioeconomic factors and variable healthcare access. We sought to examine the impact of area-level socioeconomic deprivation on access to care and outcomes for early-stage NSCLC in United States Veterans. We studied 9704 patients with clinical stage I NSCLC who underwent surgical treatment in the Veterans Health Administration (VHA) between 2006 and 2016 using a uniquely compiled database. Area-level socioeconomic deprivation was not associated with overall survival or cancer recurrence. However, high levels of socioeconomic deprivation were associated with inadequate adherence to care quality measures and increased risk of postoperative readmission. These results suggest that Veterans with high socioeconomic deprivation experience suboptimal access to quality preoperative and postoperative care for early-stage NSCLC but do not have inferior long-term outcomes following surgery. Future VHA policies should aim to provide more equitable guideline-concordant care and reduce postoperative readmission for early-stage NSCLC.

## 1. Introduction

Lung cancer remains the leading cause of cancer-related deaths in the United States (US), with over 125,000 lung cancer-related fatalities expected to occur nationwide in 2024 [1]. The incidence of early-stage non-small cell lung cancer (NSCLC) is rising across the US, which may be attributable to the increased adoption of lung cancer screening (LCS) with low-dose computed tomography (CT) and advances in imaging leading to the early detection of small tumors [2]. This nationwide shift from late- to early-stage diagnoses of NSCLC has been most pronounced within the Veteran population, which saw a 12% increase in the proportion of stage I NSCLC diagnoses from 2010 to 2017 [3,4]. Over the past decade, there has been a concurrent increase in the utilization of annual LCS among Veterans, who can receive screening through the Veterans Health Administration (VHA) with no out-of-pocket costs and have an elevated lung cancer risk due to older age, more comorbidities, high rates of smoking, and occupational exposures during military service [5,6,7,8,9]. Given that approximately 900,000 Veterans are eligible for LCS annually, the number of Veterans needing treatment for early-stage NSCLC is expected to rise significantly in the coming decade [5].

In the non-Veteran population, individual- and community-level socioeconomic factors have been shown to impact treatment rates and survival for many cancers, including lung cancer [10,11,12,13,14,15,16,17,18]. Several studies have independently found that individuals from socioeconomically deprived areas have a higher risk of developing and dying from lung cancer, which may be attributable to high rates of tobacco use, limited access to screening and treatment, and later-stage diagnosis [19,20,21,22,23,24,25,26]. In vulnerable populations, social determinants of health, such as poverty, employment opportunities, housing stability, and access to transportation, can contribute to suboptimal patient outcomes and disparities in access to cancer screening, clinical trials, molecular testing, and treatment [16,27,28,29,30]. Prior research exploring the impact of social determinants of health on lung cancer outcomes in the US has been limited by heterogeneous patient populations, variable insurance-related access to care, and, in some cases, small sample sizes [19,22,23,24,26,31]. As inadequate access to care has been linked to worse outcomes for NSCLC, it is imperative to investigate sociodemographic factors that can hinder access to care and contribute to disparities in lung cancer outcomes in the US [32].

The VHA is the nation’s largest integrated, equal-access healthcare system, with 171 medical centers and 1113 outpatient sites serving over 9 million US Veterans [33]. Eligibility for VHA benefits is determined by many factors, including length of military service, having a service-related medical condition, income, conditions of discharge from the military, and disability rating [33]. Our group has previously shown that Veterans receive high-quality care for early-stage NSCLC through the VHA with favorable outcomes compared to the civilian population, despite having a higher prevalence of serious health conditions [9]. More interestingly, the racial disparities in lung cancer outcomes observed in the US and other countries have not been observed within the Veteran population [3,18,20,28,34,35,36,37]. In fact, prior research evaluating racial differences in cancer survival within the VHA has demonstrated that Black Veterans have superior or comparable outcomes for prostate, pancreatic, laryngeal, bladder, and lung cancer, when compared to White Veterans [3,28,34,38,39,40,41,42]. While these population-level trends are encouraging, the impact of socioeconomic deprivation on lung cancer outcomes within the VHA remains unclear. The objective of the current study was to investigate the effects of area-level socioeconomic deprivation on access to care and postoperative outcomes in US Veterans undergoing surgery for stage I NSCLC. We hypothesized that Veterans with and without high socioeconomic deprivation will have comparable rates of adverse postoperative events and a similar likelihood of meeting quality measures (QMs) assessing preoperative and postoperative access to care.

## 2. Materials and Methods

### 2.1. Study Design and Population

We conducted a retrospective cohort study of patients with clinical stage I NSCLC who underwent curative-intent pulmonary resection in the VHA between 1 October 2006 and 30 September 2016. Our research protocol received approval from the Veterans Affairs St. Louis Health Care System Research and Development Committee (#1214632) and Institutional Review Board. The requirement for signed informed consent was waived due to the deidentified nature of the retrospective analyses performed. Data were reported according to the Strengthening the Reporting of Observational Studies in Epidemiology (STROBE) guidelines [43].

Data were sourced from the VHA Informatics and Computing Infrastructure (VINCI) and Corporate Data Warehouse (CDW) platforms, which integrate clinical and administrative data from various national repositories within the VHA, including CDW-Oncology Raw and the Veterans Affairs Surgical Quality Improvement Program (VASQIP). To ensure comprehensive data collection with minimal missingness, a dedicated team of research specialists (including one data analyst, two data coordinators, two statisticians, and three physicians) compiled this dataset over a two-year period using a combination of manual chart reviews and natural language processing techniques. Patients with a diagnosis of NSCLC were identified using International Classification of Diseases (ICD) for Oncology, third edition, codes. We included adults (age 18 and older) diagnosed with clinical stage I NSCLC (tumors ≤ 5 cm, node-negative), based on the American Joint Committee on Cancer’s seventh-edition staging system, who underwent definitive surgical treatment. Patients with missing diagnosis dates, those who received neoadjuvant treatment, or those with recurrent cancer were excluded from the study. Treatments and procedures were identified through query of relevant ICD-9, ICD-10, and Current Procedural Terminology (CPT) codes.

### 2.2. Area Deprivation Index and Covariates

To measure area-level socioeconomic deprivation, we assigned all patients an area deprivation index (ADI; University of Wisconsin School of Medicine and Public Health, 2019; 2015 version) score based on their residential ZIP code at the time of surgical treatment [44,45]. The ADI is a validated geographic-based metric of socioeconomic disadvantage that is calculated using seventeen different indicators of poverty, educational attainment, employment, and housing conditions determined at the census block level [17,29,32,45,46,47,48]. ADI scores range from 1 to 100 and represent national percentile rankings, with higher scores indicating higher levels of socioeconomic deprivation. The address of residence was extracted for each patient and used to assign an ADI score based on data available from the Neighborhood Atlas [44,45].

Comprehensive data on patient-, oncologic-, and treatment-related covariates were abstracted for each patient, including age, sex, body mass index, race, smoking status at time of surgery, distance lived from treatment facility (calculated from the center of the patient’s residential ZIP code), comorbidities, total number of prescription medications in the year preceding surgery, American Society of Anesthesiologists (ASA) class, tumor location, size, grade, histology, year of operation, annual hospital case volume (defined as the volume of lung cancer cases treated at a specific facility in the year preceding surgery), preoperative pulmonary function testing (PFT) results (based on forced expiratory volume in one second), surgical approach, lung resection type, intraoperative lymph node sampling assessment (≥three N2 and one N1 nodal stations), surgical margins, and pathologic stage. Race was categorized as Black, White, or other based on coding in the CDW and as defined by the American College of Surgery Facility Oncology Registry Data Standards. Patient comorbidities were assessed using the composite Charlson–Deyo Comorbidity Index, which was derived using ICD-9 and ICD-10 codes recorded from five years prior to one month after surgery [49,50].

### 2.3. Outcomes and Care Quality Metrics

The primary outcome of this study was adherence to QMs assessing preoperative and postoperative access to care for NSCLC. Upon review of contemporary treatment guidelines from the American College of Chest Physicians, European Society for Medical Oncology, and National Comprehensive Cancer Network (NCCN), our group previously defined four QMs for preoperative care in stage I NSCLC: timely surgery, positron emission tomography (PET) imaging, appropriate smoking management, and PFT [32]. Adherence to all four of these preoperative care QMs has been associated with a lower likelihood of postoperative mortality and improved long-term survival [32]. In the current study, timely surgery was defined as undergoing curative-intent lung resection within twelve weeks of radiographic diagnosis [29]. The receipt of PET imaging was defined as having a PET scan performed within six months prior to the date of surgery and was evaluated via query of relevant CPT codes. Appropriate smoking management was determined by whether individuals who smoked received suitable smoking cessation pharmacotherapy (nicotine replacement therapy [NRT], bupropion, or varenicline) within 12 months preceding surgery [7]. Our group has previously highlighted the underutilization of guideline-recommended smoking cessation treatments during the perioperative period, which is linked to adverse outcomes [7]. Prescription details for these medicines were obtained from the CDW Pharmacy Outpatient database, using methodology previously described by our group [50]. Individuals who were either former or never smokers were recorded as successfully meeting this QM. The receipt of PFTs was defined as having undergone spirometry evaluation within six months prior to surgery based on review of CPT codes.

Similarly, we established three guideline-concordant postoperative QMs that patients with pathological stage I NSCLC (tumors ≤ 5 cm and N0 on surgical pathology report) should routinely meet postoperatively: appropriate surveillance imaging, appropriate smoking management, and appropriate referral to medical oncology. Appropriate surveillance imaging was characterized as having at least one CT scan annually during the first two years following surgery. Previous research from our group has shown that optimal surveillance imaging should be conducted at least annually in the early postoperative period [51,52]. Appropriate smoking management was defined as being either a never smoker, former smoker, or a current smoker who received suitable smoking cessation pharmacotherapy (NRT, bupropion, or varenicline) within the year after surgery. Appropriate oncology referral was defined as receiving a medical oncology referral (according to VHA visit codes) within six months after surgery for patients with tumors > 3 cm or high-risk clinicopathologic features, as recommended by the NCCN [53]. Patients with tumors < 3 cm without high-risk clinicopathologic features were considered appropriately managed if they were not referred to medical oncology. Examples of high-risk clinicopathologic features include poorly differentiated tumors, vascular invasion, wedge resection, visceral pleural involvement, and unknown lymph node status [53].

We also assessed multiple secondary outcomes, including prolonged hospital length of stay (LOS; defined as ≥14 days), 30-day postoperative readmission, 30-day major postoperative complications, 30- and 90-day mortality, overall survival (OS), and cumulative incidence of cancer recurrence. Major complications were defined as occurrences of respiratory failure, empyema, pneumonia, stroke, myocardial infarction, or renal failure within 30 days following surgery. These complications were detected through query of the VASQIP database, a validated tool for identifying postoperative complications, as well as ICD-9 and ICD-10 diagnosis codes [9,28,50,54,55]. The date of death was ascertained for patients using the VHA Vital Status Files. OS data were censored at the end of the study’s follow-up period, which concluded on 1 May 2020. Cancer recurrence was evaluated using the CDW Oncology database and was further supplemented by identifying additional diagnoses indicative of recurrence through ICD-9 and ICD-10 codes, as previously outlined by our research team and in the VHA literature [8,29,50,56].

### 2.4. Statistical Analysis

Univariate restricted cubic spline (RCS) analyses were conducted to model the relationship between ADI and the primary and secondary outcomes. RCS models are advantageous as they effectively identify threshold values for significant predictor variables without assuming a specific shape of the function [29,57]. Splines create nonlinear models that link a continuous independent variable (e.g., ADI) with a dependent outcome (e.g., 30-day mortality) without the need to categorize the independent variable, thereby preserving statistical power that is often lost with categorization [58]. The resulting curve shapes were analyzed to determine if there was an inflection point where the likelihood of each outcome changed significantly. Select univariate RCS model curves are provided in Supplemental Figure S1. Based on the RCS analyses, differences in the likelihood of most outcomes were noted to occur around ADI scores of 50 and 75. Hence, we categorized patients using their ADI measure into three groups corresponding to differing levels of socioeconomic deprivation: ADI ≤ 50 (low), 50 < ADI ≤ 75 (moderate), and ADI > 75 (high).

The association of ADI with meeting all QMs and postoperative outcomes was assessed using multivariable logistic regression models after adjusting for relevant demographic, comorbidity, oncologic, and operative characteristics. OS was evaluated using the multivariable Cox proportional hazard model and was presented using the Kaplan–Meier method. Cumulative incidence of cancer recurrence was assessed with a multivariable competing risk model (Fine–Gray subdistribution hazard model), with cancer recurrence as the outcome and mortality as a competing event.

Descriptive statistics were presented as means with standard deviations (SDs) for continuous variables and as frequencies with corresponding proportions for categorical variables. Median values were displayed for nonparametric covariates. Comparisons of continuous variables with a normal distribution were performed using Student’s *t*-test. The Mann–Whitney U test was used to compare nonparametric variables when appropriate. The Kolmogorov–Smirnov test was applied to test if data were normally distributed. Categorical variables were compared using Pearson’s chi-squared test. However, if one or more groups had too few observations, Fisher’s exact test was applied instead. Missing data were minimal and were classified as unknown. All statistical tests were conducted as two-sided, with *p*-values < 0.05 considered statistically significant. The analyses were performed using SAS version 9.3 (SAS Institute Inc., Cary, NC, USA).

## 3. Results

In total, 9704 Veterans who underwent surgery for clinical stage I NSCLC met our inclusion criteria. The mean age was 67.6 (SD: 7.9) years (Table 1). Most patients were male (n = 9348; 96.3%), of White race (n = 8027; 82.7%), with multiple medical comorbidities (Charlson–Deyo Comorbidity Index score, mean: 6.88; SD: 2.21), were smoking at the time of surgery (n = 5682; 58.6%), and lived ≥11 miles from their treatment facility (n = 7590; 78.2%). Tumor histology was most frequently adenocarcinoma (n = 5168; 53.3%). The most common type of pulmonary resection was lobectomy (n = 6873; 70.8%), and most patients underwent a thoracotomy (n = 5651; 58.4%). The rates of R0 resection and pathologic upstaging were 96.7% (n = 9286) and 12.9% (n = 1250), respectively.

The median ADI score for the study cohort was 61 (interquartile range [IQR]: 43–76). Of 9704 total Veterans, 3272 (33.7%) had an ADI score of ≤50 (low), 3885 (40.0%) had a score of 50 < ADI ≤ 75 (moderate), and 2547 (26.3%) had an ADI score of >75 (high). In multivariable linear analysis, high ADI was associated with younger age, higher ASA class, increased annual hospital case volume, squamous cell carcinoma, left lower lobe tumors, and tumors >2 cm (all *p* < 0.05; Supplemental Table S1).

### 3.1. Preoperative Care Quality Measures

**Figure 1:**
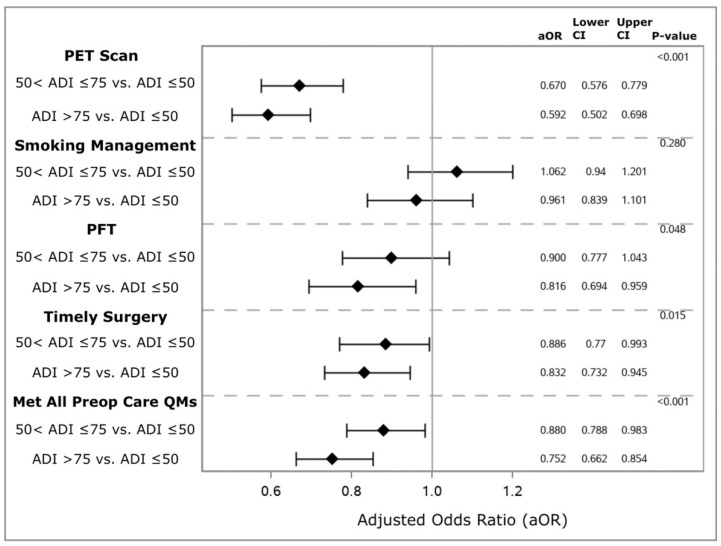
Association between area deprivation index and adherence to quality metrics assessing access to preoperative care. Models adjust for area deprivation index score, age, race, sex, body mass index, smoking status at surgery, Charlson–Deyo Comorbidity Index score, American Society of Anesthesiologists class, preoperative forced expiratory volume in one second, number of prescription medications in the year prior to surgery, distance lived from treatment facility, annual hospital case load, tumor histology, tumor grade, tumor location, tumor size, year of operation. Abbreviations used: ADI—area deprivation index; aOR—adjusted odds ratio; CI—95% confidence interval; PET—positron emission tomography; PFT—pulmonary function testing; Preop—preoperative; QMs—quality measures.

In terms of preoperative care, 42 (0.43%) Veterans met zero QMs, 442 (4.6%) Veterans met one QM, 1847 (19.0%) Veterans met two QMs, 4021 (41.4%) met three QMs, and 3352 (34.5%) Veterans met all four QMs. In total, 6674 (68.8%) Veterans underwent timely surgery, 8301 (82.8%) received PET imaging before surgery, 6852 (70.6%) received appropriate smoking management, and 8050 (83.0%) had PFT performed prior to surgery. Compared with Veterans residing in areas with low socioeconomic deprivation, those from areas with high socioeconomic deprivation had a reduced likelihood of receiving PET imaging (multivariable-adjusted odds ratio [aOR] 0.592, 95% confidence interval (CI) 0.502–0.698) and PFT (aOR 0.816, 95% CI 0.694–0.959) as well as having timely surgery (aOR 0.832, 95% CI 0.732–0.945) (Figure 1). There was no association between high ADI and receiving appropriate preoperative smoking management (aOR 0.961, 95% CI 0.839–1.101). Veterans from areas of moderate (aOR 0.880, 95% CI 0.788–0.983) and high (aOR 0.752, 95% CI 0.662–0.854) socioeconomic deprivation had lower risk-adjusted odds of meeting all four QMs (yes versus no) compared to those from areas of low socioeconomic deprivation.

### 3.2. Postoperative Outcomes

**Figure 2:**
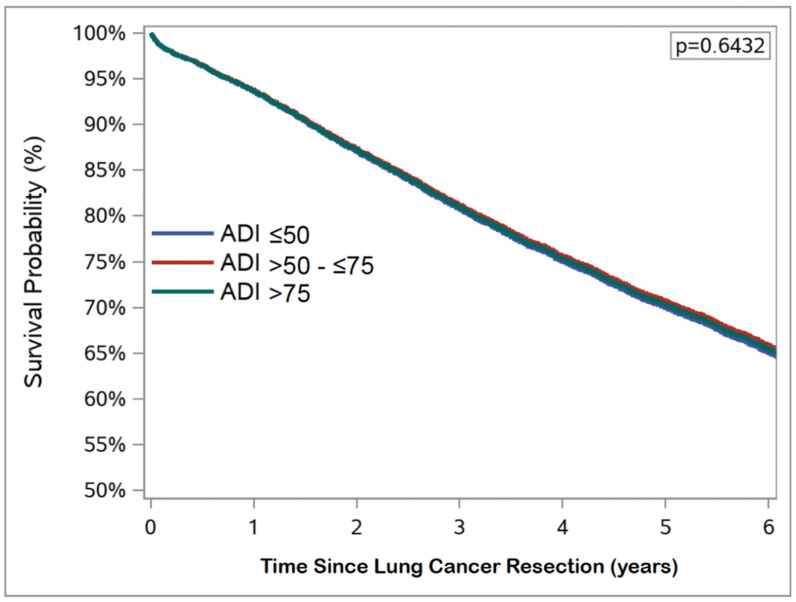
Adjusted Kaplan–Meier survival analysis stratified by area deprivation index (ADI) score.

**Figure 3:**
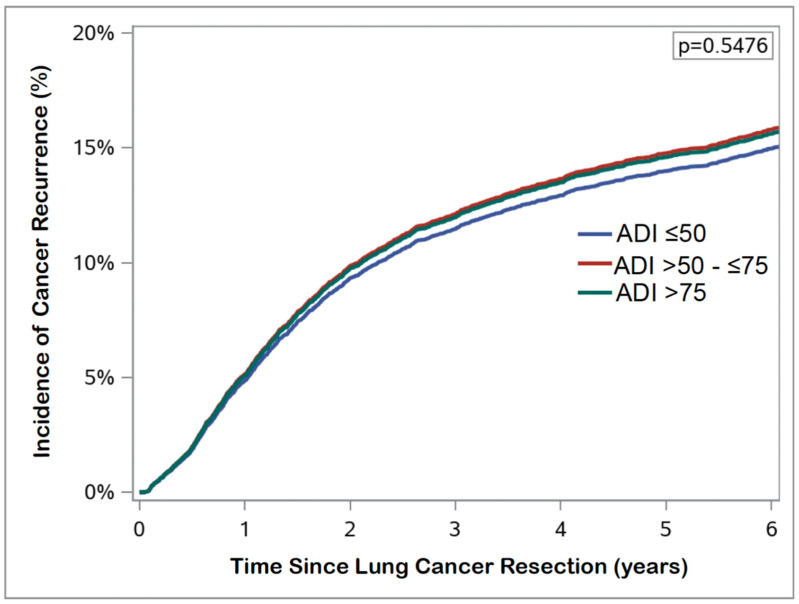
Fine–Gray competing risk analysis examining cumulative incidence of cancer recurrence based on area deprivation index (ADI) score.

In the study cohort, prolonged hospital LOS occurred in 1403 (15.4%) patients. Major postoperative complications within 30 days occurred in 1343 (13.8%) patients. The rates of 30-day postoperative mortality, 30-day readmission, and 90-day postoperative mortality were 2.1% (n = 206), 7.8% (n = 758), and 4.0% (n = 388), respectively. In multivariable risk-adjusted analyses, Veterans from areas with high socioeconomic deprivation had increased odds of 30-day readmission (aOR 1.380, 95% CI 1.103–1.726) compared to those from areas of low socioeconomic deprivation. There was no significant difference in risk of prolonged hospital LOS, 30-day major complications, or 30- and 90-day postoperative mortality between groups (Table 2). Similarly, moderate (adjusted hazard ratio [aHR] 0.968, 95% CI 0.904–1.036) and high (aHR 0.984, 95% CI 0.911–1.062) socioeconomic deprivation were not associated with OS after risk-adjustment (Figure 2). There was also no association between moderate (aHR 1.060, 95% CI 0.953–1.180) or high (aHR 1.047, 95% CI 0.930–1.179) socioeconomic deprivation and cumulative incidence of cancer recurrence (Figure 3).

### 3.3. Postoperative Care Quality Measures

**Figure 4:**
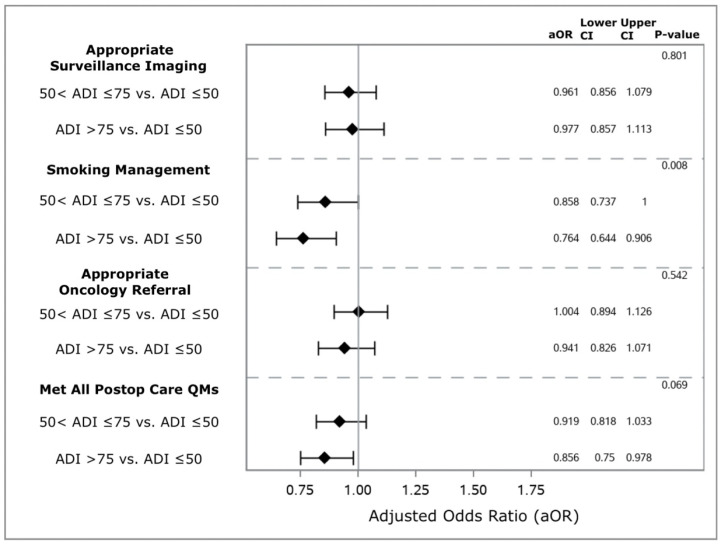
Association between area deprivation index and adherence to quality metrics assessing access to postoperative care. Models adjust for area deprivation index score, age, race, sex, body mass index, smoking status at surgery, Charlson–Deyo Comorbidity Index score, American Society of Anesthesiologists class, preoperative forced expiratory volume in one second, number of prescription medications in the year prior to surgery, distance lived from treatment facility, annual hospital case load, tumor histology, tumor grade, tumor location, tumor size, year of operation, surgical approach, lung resection type, adequate intraoperative lymph node sampling, adherence to all preoperative care quality measures (yes versus no). Abbreviations used: ADI—area deprivation index; aOR—adjusted odds ratio; CI—95% confidence interval; Postop—postoperative; QMs—quality measures.

In total, 8111 (95.9%) of 8454 Veterans with pathological stage I NSCLC had complete follow-up data available and were evaluated for adherence to postoperative care QMs. Of these Veterans, 368 (4.5%) met zero QMs, 2087 (25.7%) met one QM, 2187 (27.0%) Veterans met two QMs, and 3469 (42.8%) met all three QMs. Overall, 4342 (53.5%) Veterans underwent appropriate postoperative surveillance imaging, 6514 (80.3%) received appropriate postoperative smoking management, and 4318 (53.2%) received an appropriate referral to medical oncology. Compared with Veterans from areas of low socioeconomic deprivation, those from areas of high socioeconomic deprivation had similar risk-adjusted odds of receiving appropriate surveillance imaging (aOR 0.977, 95% CI 0.857–1.113; Figure 4) and appropriate medical oncology referral (aOR 0.941, 95% CI 0.826–1.071). However, Veterans with high ADI were less likely to receive appropriate smoking management (aOR 0.764, 95% CI 0.644–0.906). Veterans from areas with moderate socioeconomic deprivation had similar risk-adjusted odds of meeting all three postoperative care QMs (yes versus no) compared to those from areas with low socioeconomic deprivation (aOR 0.919, 95% CI 0.818–1.033). Meanwhile, Veterans from areas with high socioeconomic deprivation had a significantly lower likelihood of meeting all three QMs (yes versus no; aOR 0.856, 95% CI 0.750–0.978).

## 4. Discussion

In the present study, we evaluated the relationship between socioeconomic deprivation and adherence to guideline-concordant care QMs in patients with stage I NSCLC, in an integrated healthcare system that theoretically offers equitable access to care. Consistent with previously reported findings from Samson et al. and Heiden et al., we noted that the proportion of Veterans meeting all QMs for stage I NSCLC was suboptimal in both the preoperative (34.5%) and postoperative (42.8%) periods [32,59]. Although we found that Veterans from areas with high socioeconomic deprivation were less likely to meet preoperative and postoperative care QMs, compared to their counterparts residing in more affluent areas, short- and long-term postoperative outcomes were largely similar between groups. Collectively, these findings suggest that while the VHA may mitigate the impact of socioeconomic deprivation on long-term outcomes after curative resection for stage I NSCLC, there are significant opportunities to address gaps in access to care QMs in the Veteran population.

Data on area-level socioeconomic conditions are valuable for health services researchers and oncology providers as they can reflect individual socioeconomic status and the broader environment in which cancer patients live [17,23,24,26,46,48,60,61]. Residents of economically disadvantaged or rural areas often experience adverse health outcomes, which may be due to limited healthcare access or inadequate infrastructure to support cancer care in these regions [22,23,25,37,46,47,60]. When investigating cancer disparities, epidemiologists and health services researchers must recognize the role of neighborhood conditions, which are influenced by the distribution of race, ethnicity, and socioeconomic class, in shaping lung cancer outcomes. Unfortunately, valuable data on area-based socioeconomic measures are frequently missing from population-based cancer registries [61,62]. Our findings underscore that where lung cancer patients live may partly account for disparities in treatment and outcomes. Hence, public health experts and policy makers should be cognizant of the significance of individuals’ living environments when developing new policies and targeted interventions aimed at improving lung cancer outcomes among marginalized populations.

Neighborhood-level sociodemographic determinants have been associated with lung cancer incidence and mortality in multiple countries, including the US, independent of individual-level characteristics [19,20,24,31,35,36,37,63]. While prior research has primarily focused on heterogenous populations and healthcare systems, the current study uniquely examines the effects of socioeconomic disadvantage on lung cancer outcomes in the VHA, the largest integrated and equal-access healthcare system in the US. Previous research from our group and others has found that the quality of care provided by VHA hospitals is comparable or superior to the care provided by non-VHA hospitals [9,64,65]. In a propensity score-matched study using national data from the VHA and National Cancer Database, Heiden and colleagues showed that Veterans undergoing surgical treatment for clinical stage I NSCLC had significantly lower postoperative mortality and better OS than the non-Veteran population [9]. These findings may be partly attributable to concerted efforts within the VHA to mitigate the effects of social determinants of health among Veterans receiving lung cancer care. Today, VHA hospitals screen patients for a wide array of health-related social needs including food insecurity, substance abuse, inadequate transportation, housing instability, intimate partner violence, legal services, and social isolation [66,67,68,69,70]. Furthermore, health equity remains a core principle of the VHA mission and is continuously assessed by several internal committees including the Center for Health Equity Research and Promotion, the Health Equity and Rural Outreach Innovation Center, and the Office of Health Equity [3]. Quality improvement initiatives focused on mitigating the impact of social determinants of health, such as screening and referral programs for health-related social needs and free transportation services, have contributed to the VHA’s ability to reduce certain disparities in cancer care among the Veteran population [3,28,34].

A notable finding from this study is the lack of observed association between area-level socioeconomic deprivation and the risk of short- or long-term mortality after lung cancer resection. Several authors have previously reported that socioeconomic determinants can significantly impact lung cancer survival among the non-Veteran population [19,22,23,28,31]. In one study from the Duke University Health System, Erhunmwunsee and colleagues found that low socioeconomic status was an independent prognostic factor for reduced survival in patients with early- and advanced-stage NSCLC [31]. Similarly, Shugarman et al. demonstrated that individual and regional socioeconomic factors are associated with a higher risk of lung cancer mortality among Medicare beneficiaries [71]. In contrast to these findings, our group and others have shown that lung cancer survival is improving within the VHA, with Black Veterans experiencing similar or better survival rates than White Veterans [3,28,34]. Similarly, our study demonstrated that Veterans residing in areas with high concentrations of socioeconomic deprivation have similar long-term survival and risk of cancer recurrence compared to Veterans residing in more affluent areas. This trend has not been observed in the broader US population and could be attributed to VHA patients generally having more equitable access to care than those with private insurance. Through the VHA, Veterans receive free transportation services to medical appointments, referrals for health-related social needs, and access to medications, surgeries, clinic visits, and rehabilitation services with often minimal to no out-of-pocket costs [28,50,72,73,74]. The VHA’s success in achieving equitable lung cancer survival outcomes across a diverse patient population with high rates of comorbidities requires further study to better understand how this accomplishment might be replicated in other healthcare systems [9].

While the VHA has made significant strides in ensuring healthcare equity, our study found that Veterans with stage I NSCLC who live in socioeconomically deprived areas continue to experience barriers to accessing guideline-concordant care and have a higher risk of readmission after curative-intent resection. The decreased odds of meeting QMs in stage I NSCLC care among socioeconomically disadvantaged Veterans might be explained by the presence of structural and environmental barriers. Individual- and neighborhood-level socioeconomic factors have been shown to influence treatment rates for multiple cancers [10,11,14,15,16]. Veterans residing in impoverished areas may experience barriers to accessing critical components of lung cancer care, such as preoperative PET imaging and timely surgical evaluation, due to limited access to nearby hospitals, public transportation systems, financial resources, social support, and stable housing. Hence, addressing the health-related social needs of Veterans should be recognized as a key component of high-quality lung cancer care, as it directly impacts treatment outcomes. In an effort to mitigate existing disparities in lung cancer care, future VHA policies should continue to focus on the development of targeted initiatives to improve the delivery of equitable and guideline-concordant perioperative care for early-stage NSCLC.

This study has several important strengths. Most importantly, it utilizes a nationally representative and comprehensive dataset that includes an extensive list of treatment-related covariates with high accuracy and minimal missing data. Additionally, the dataset was meticulously curated by a dedicated research team to ensure strict data quality. This study also uniquely reports on adherence to established preoperative and postoperative care QMs for stage I NSCLC care, which is notable as most contemporary oncology registries do not include granular details about care QMs. However, there are also limitations to this work. First, the VHA primarily serves a distinct patient population, which frequently includes men, of White race, with high comorbidity burdens and rates of smoking. Given the distinctive features of the Veteran population and the VHA system, further research is needed to determine if our findings are broadly applicable to the non-Veteran population with early-stage NSCLC. Second, this study uses the ADI, which is a validated measure of neighborhood socioeconomic deprivation at the census block group level, though it may not fully capture each patient’s individual socioeconomic status. Lastly, it is possible that some aspects of preoperative and postoperative care may have been provided outside the VHA system and thus were not available for abstraction from the VHA data repositories. However, prior work from our group suggests that most Veterans receive the majority, if not all, of their lung cancer care within the VHA, as exhibited by their multiple encounters with VHA facilities before and after surgery, reducing the likelihood of this bias [7,32,50].

## 5. Conclusions

Collectively, our findings suggest that Veterans with high socioeconomic deprivation have suboptimal adherence to care QMs for early-stage NSCLC but do not have inferior long-term outcomes after curative-intent resection. While the VHA healthcare model may mitigate the impact of socioeconomic deprivation on survival for resectable stage I NSCLC, there are still substantial opportunities to improve gaps in access to quality care for this patient population. Future VHA policies should aim to increase adherence to guideline-concordant QMs and reduce postoperative readmission for socioeconomically disadvantaged Veterans with early-stage NSCLC. Lastly, the VHA’s model of integrated, equal-access healthcare may serve as a useful framework for other healthcare systems seeking to enhance their ability to address disparities in lung cancer mortality across diverse populations.

## Figures and Tables

**Table 1:** Demographics of study population.

Demographics	Study Population, n = 9704 (%)
Age, **MEAN (STANDARD DEVIATION [SD])**	67.60 (7.89)
Sex	
Male	9348 (96.33)
Female	356 (3.67)
Race	
White	8027 (82.72)
Black	1453 (14.97)
Other	131 (1.35)
Unknown	93 (0.96)
Body mass index **(N = 126 MISSING)**	
<18.5	307 (3.21)
18.5–24.9	3265 (34.09)
25–29.9	3445 (35.97)
30–34.9	1830 (19.11)
≥35	731 (7.63)
Smoking status at surgery	
Never	132 (1.36)
Former	3890 (40.09)
Current	5682 (58.55)
Charlson–Deyo Comorbidity Index score, **MEAN (SD)**	6.88 (2.1)
Total number of medications prescribed in the year prior to surgery, **MEAN (SD)**	13.71 (7.93)
American society of anesthesiologists (ASA) class, **(N = 404 MISSING)**	
1	0 (0.0)
2	333 (3.58)
3	7418 (79.76)
4	1549 (16.66)
5	0 (0.0)
Predicted forced expiratory volume in 1 s **(N = 497 MISSING)**	
<50%	619 (6.72)
50–79%	4249 (46.15)
≥80%	4339 (47.13)
Tumor lobe location **(N = 124 MISSING)**	
Right upper	3520 (36.74)
Left upper	2654 (27.70)
Right lower or middle	2066 (21.57)
Left lower	1340 (13.99)
Area Deprivation Index score, median (IQR)	61 (43–76)
Distance from treatment facility **(MILES)**	
0–10	2114 (21.78)
11–50	3913 (40.32)
>50	3677 (37.89)
Annual hospital volume, **MEAN (SD)**	100.64 (53.39)
Tumor size **(MM; N = 6 MISSING)**	
≤10	888 (9.15)
11–20	3907 (40.26)
21–30	2679 (27.61)
31–40	1496 (15.42)
41–50	728 (7.50)
Tumor grade **(N = 567 MISSING)**	
I	1206 (13.20)
II	4815 (52.70)
III	2984 (32.66)
IV	132 (1.44)
Tumor histology	
Adenocarcinoma	5168 (53.26)
Squamous cell carcinoma	3281 (33.81)
Other	1255 (12.93)
Adequate intraoperative lymph node sampling	
≥three N2 and one N1 nodal stations	2556 (26.34)
<three N2 and one N1 nodal stations	7148 (73.66)
Surgical approach **(N = 26 MISSING)**	
Video-assisted thoracoscopic surgery	4027 (41.61)
Thoracotomy	5651 (58.39)
Lung resection type	
Lobectomy	6873 (70.83)
Wedge	2138 (22.03)
Segmentectomy	538 (5.54)
Pneumonectomy	155 (1.60)
Pathologic upstaging	
Present	1250 (12.88)
Absent	8454 (87.12)
Surgical resection margin	
R0	9286 (96.70)
R1 OR R2	317 (3.30)

**Table 2:** Association between area deprivation index (ADI) score and postoperative outcomes *.

Postoperative Outcome	Adjusted Odds/Hazard Ratio (95% Confidence Interval)	*p*-Value
**Prolonged Hospital Length of Stay**		0.405
ADI ≤ 50	Reference	
50 < ADI ≤ 75	0.937 (0.807–1.088)	
ADI > 75	0.893 (0.755–1.056)	
**30-day Readmission**		0.019
ADI ≤ 50	Reference	
50 < ADI ≤ 75	1.173 (0.955–1.441)	
ADI > 75	1.380 (1.103–1.726)	
**30-day Major Complications**		0.274
ADI ≤ 50	Reference	
50 < ADI ≤ 75	1.056 (0.908–1.228)	
ADI > 75	0.927 (0.780–1.101)	
**30-day Mortality**		0.445
ADI ≤ 50	Reference	
50 < ADI ≤ 75	0.970 (0.662–1.421)	
ADI > 75	1.221 (0.816–1.826)	
**90-day Mortality**		0.699
ADI ≤ 50	Reference	
50 < ADI ≤ 75	0.943 (0.720–1.237)	
ADI > 75	0.876 (0.645–1.190)	
**Overall Survival**		0.643
ADI ≤ 50	Reference	
50 < ADI ≤ 75	0.968 (0.904–1.036)	
ADI > 75	0.984 (0.911–1.062)	
**Cumulative Incidence of Cancer Recurrence**		0.548
ADI ≤ 50	Reference	
50 < ADI ≤ 75	1.060 (0.953–1.180)	
ADI > 75	1.047 (0.930–1.179)	

* Models adjust for area deprivation index score, age, race, sex, body mass index, smoking status at surgery, Charlson–Deyo Comorbidity Index score, American Society of Anesthesiologists class, preoperative forced expiratory volume in one second, number of prescription medications in the year prior to surgery, distance lived from treatment facility, annual hospital case load, tumor histology, tumor grade, tumor location, tumor size, year of operation, surgical approach, lung resection type, adequate intraoperative lymph node sampling, pathological upstaging, adherence to all preoperative care quality measures (yes versus no). Complete results from the regression models evaluating the relationship between area deprivation index and postoperative outcomes are detailed in Supplemental Tables S2–S8.

## Data Availability

The patient-level data used in this study are maintained by the United States Department of Veterans Affairs (VA). These data are freely available to VA-affiliated researchers with VA-secured computing access after appropriate study protocol approval. For more information, visit https://www.virec.research.va.gov or contact the VA Information Resource Center at VIReC@va.gov. Additional inquiries can be directed to the corresponding author. Deidentified data from the VA study population discussed in this article can be made available upon request with appropriate Institutional Review Board and VA approval as well as data use agreements. We may balance the potential benefits and risks of each request and then provide the data that can be shared.

## Supplementary Materials

The following supporting information can be downloaded at https://www.mdpi.com/article/10.3390/cancers16223788/s1, Figure S1: Select univariate restricted cubic spline models exhibiting the relationship between area deprivation index with postoperative outcomes and meeting care quality metrics; Table S1: Multivariable analysis of factors associated with high area deprivation index score; Table S2: Results from multivariable regression model constructed to evaluate the relationship between area deprivation index and prolonged hospital length of stay; Table S3: Results from multivariable regression model constructed to evaluate the relationship between area deprivation index and 30-day hospital readmission; Table S4: Results from multivariable regression model constructed to evaluate the relationship between area deprivation index and 30-day major postoperative complications; Table S5: Results from multivariable regression model constructed to evaluate the relationship between area deprivation index and 30-day postoperative mortality; Table S6: Results from multivariable regression model constructed to evaluate the relationship between area deprivation index and 90-day postoperative mortality; Table S7: Results from multivariable regression model constructed to evaluate the relationship between area deprivation index and overall survival; Table S8: Results from multivariable regression model constructed to evaluate the relationship between area deprivation index and cumulative incidence of cancer recurrence.
