# Supplementary material for: Impact of Socioeconomic Deprivation on Care Quality and Surgical Outcomes for Early-Stage Non-Small Cell Lung Cancer in United States Veterans
Source: Cancers (Basel). 2024 Nov 11;16(22):3788. doi: 10.3390/cancers16223788 (PMC11593132; doi:10.3390/cancers16223788)
Supplement: Supplementary file 1 [file cancers-16-03788-s001.zip › cancers-3258946-supplementary.pdf]

Supplementary Materials

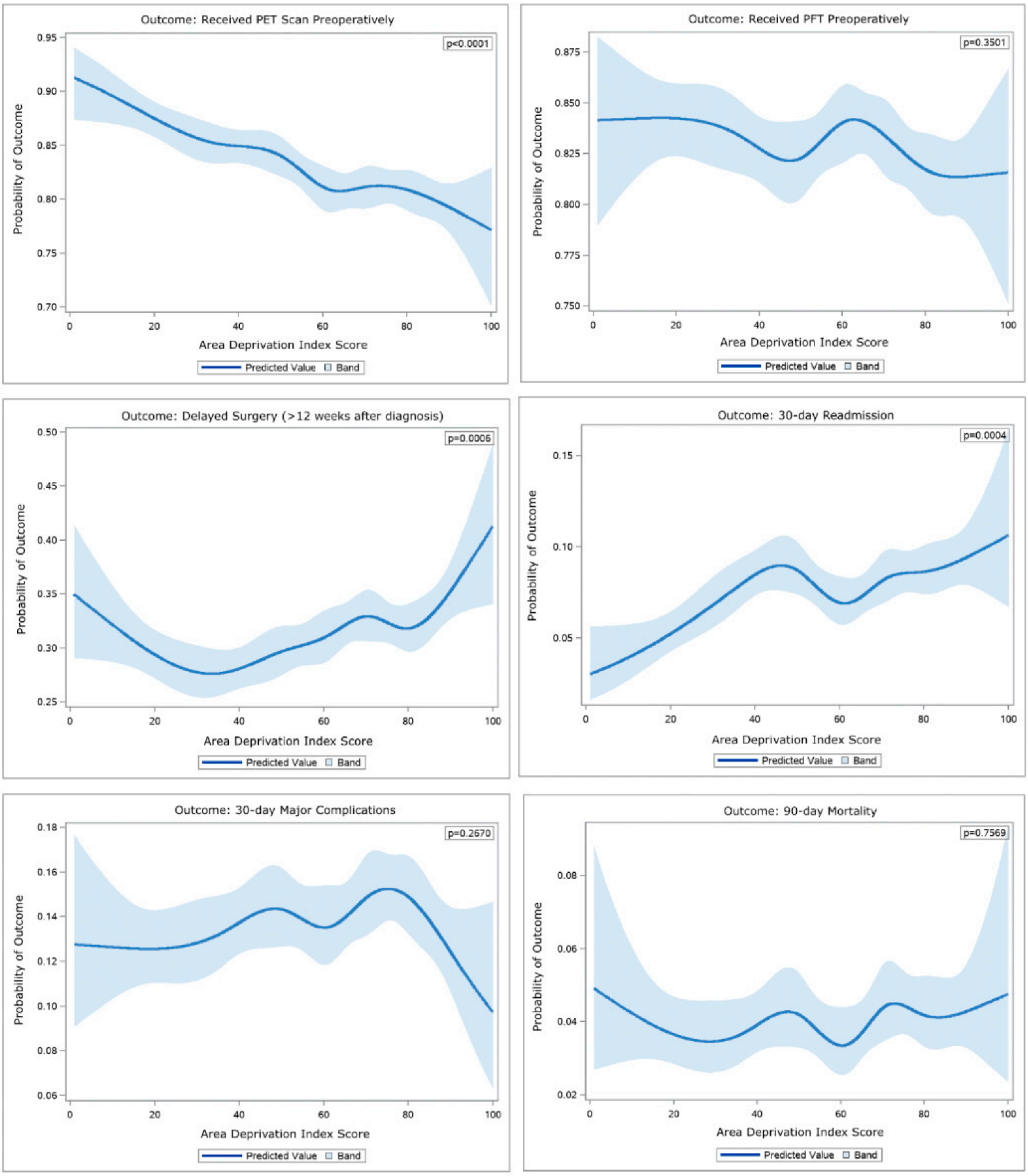

Supplemental Figure S1. Select univariate restricted cubic spline models exhibiting the relationship between area deprivation index with postoperative outcomes and meeting care quality metrics.

Supplemental Table S1. Multivariable analysis of factors associated with high area deprivation index score.

Parameter	Estimate	Standard Error	T-value	P-value
Intercept	77.857	112.896	0.69	0.490
Age	-0.143	0.024	-5.84	<0.001
Sex *(reference: Male)*				
Female	-0.955	0.849	-1.12	0.261
Race *(reference: White)*				
Black	0.009	0.468	0.02	0.985
Other	-1.183	1.347	-0.88	0.380
Unknown	-4.360	1.649	-2.64	0.008
Body mass index *(reference: 18.5-24.9)*				
<18.5	1.391	0.930	1.50	0.135
25-29.9	0.251	0.385	0.65	0.515
30-34.9	-0.077	0.466	-0.17	0.869
≥35	0.065	0.657	0.10	0.921
Smoking status at surgery *(reference: Current)*				
Never	-0.294	1.385	-0.21	0.832
Former	-0.192	0.338	-0.57	0.571
Charlson-Deyo Comorbidity Index score	0.080	0.090	0.90	0.371
American Society of Anesthesiologists Class	2.344	0.384	6.10	<0.001
Preoperative forced expiratory volume (FEV) in 1 second *(reference: FEV1 ≥80% predicted)*				
FEV1 50-79% predicted	0.142	0.336	0.42	0.672
FEV1 <50% predicted	0.836	0.666	1.26	0.209
Number of prescription medications in the year before surgery	0.014	0.022	0.62	0.538
Distance from treatment facility *(reference: 0-10 miles)*				
11-50 miles	1.536	0.432	3.55	<0.001
>50 miles	9.834	0.440	22.34	<0.001
Annual hospital case volume	0.020	0.003	6.69	<0.001
Tumor size *(reference ≤10 mm)*				
11-20 mm	1.036	0.587	1.76	0.078
21-30 mm	1.314	0.614	2.14	0.033
31-40 mm	1.726	0.676	2.55	0.011
41-50 mm	1.899	0.800	2.37	0.018
Tumor grade *(reference: I)*				
II	0.828	0.505	1.64	0.101
III	0.568	0.544	1.04	0.296
IV	1.311	1.436	0.91	0.361
Tumor histology *(reference: Adenocarcinoma)*				
Squamous cell carcinoma	0.735	0.364	2.02	0.043
Other	-0.753	0.529	-1.42	0.155
Tumor location *(reference: Right upper lobe)*				
Left lower lobe	1.229	0.503	2.44	0.015
Left upper lobe	0.152	0.401	0.38	0.704
Right lower or middle lobe	0.219	0.435	0.50	0.614
Year of operation	-0.016	0.056	-0.28	0.782

Supplemental Table S2. Results from multivariable regression model constructed to evaluate the relationship between area deprivation index and prolonged hospital length of stay.

Parameter	Adjusted Odds Ratio	Lower Limit of 95% Confidence Interval	Upper Limit of 95% Confidence Interval	P-value
Area deprivation index (ADI) score *(reference: ADI ≤50)*				0.4051
50< ADI ≤75	0.937	0.807	1.088	
ADI >75	0.893	0.755	1.056	
Age	1.012	1.002	1.021	0.0148
Sex *(reference: Male)*				0.7683
Female	0.947	0.662	1.357	
Race *(reference: White)*				0.5710
Black	0.933	0.777	1.120	
Other	0.718	0.387	1.334	
Unknown	1.211	0.652	2.250	
Body mass index *(reference: 18.5-24.9)*				<0.0001
<18.5	1.436	1.049	1.966	
25-29.9	0.715	0.617	0.828	
30-34.9	0.514	0.424	0.623	
≥35	0.508	0.385	0.670	
Smoking status at surgery *(reference: Current)*				0.4064
Never	0.754	0.385	1.478	
Former	1.069	0.936	1.220	
Charlson-Deyo Comorbidity Index score	1.075	1.040	1.112	<0.0001
American Society of Anesthesiologists Class	1.393	1.205	1.610	<0.0001
Preoperative forced expiratory volume (FEV) in 1 second *(reference: FEV1 ≥80% predicted)*				0.0032
FEV1 50-79% predicted	1.138	0.997	1.299	
FEV1 <50% predicted	1.501	1.175	1.917	
Number of prescription medications in the year before surgery	1.017	1.008	1.026	0.0001
Distance from treatment facility *(reference: 0-10 miles)*				0.0450
11-50 miles	0.811	0.687	0.956	
>50 miles	0.882	0.743	1.046	
Annual hospital case volume	0.999	0.998	1.000	0.0469
Tumor size *(reference ≤10 mm)*				0.2308
11-20 mm	0.892	0.707	1.126	
21-30 mm	0.798	0.625	1.019	
31-40 mm	0.878	0.672	1.146	
41-50 mm	1.005	0.741	1.362	
Unknown	3.765	0.306	46.302	
Tumor grade *(reference: I)*				0.3265
II	1.037	0.840	1.281	
III	1.156	0.926	1.443	
IV	0.895	0.502	1.597	
Tumor histology *(reference: Adenocarcinoma)*				0.0030

Squamous cell carcinoma	1.271	1.105	1.461	
Other	1.186	0.967	1.456	
Tumor location *(reference: Right upper lobe)*				<0.0001
Left lower lobe	0.635	0.515	0.782	
Left upper lobe	0.896	0.768	1.044	
Right lower or middle lobe	0.756	0.636	0.898	
Surgical approach *(reference: Thoracotomy)*				<0.0001
Minimally invasive	0.522	0.451	0.604	
Lung resection type *(reference: Lobectomy)*				<0.0001
Pneumonectomy	1.200	0.776	1.855	
Segmentectomy	0.747	0.556	1.005	
Wedge resection	0.589	0.493	0.704	
Adequate lymph node sampling *(reference: <three N2 and one N1 nodal stations)*				0.8379
≥three N2 and one N1 nodal stations	0.985	0.853	1.138	
Year of operation	0.971	0.949	0.994	0.0129
Pathologic Upstaging Present *(reference: No)*				0.0749
Yes	1.183	0.983	1.422	
Met All Preoperative Care Quality Measures *(reference: No)*				0.3166
Yes	0.933	0.815	1.069	

Supplemental Table S3. Results from multivariable regression model constructed to evaluate the relationship between area deprivation index and 30-day hospital readmission.

Parameter	Adjusted Odds Ratio	Lower Limit of 95% Confidence Interval	Upper Limit of 95% Confidence Interval	P-value
Area deprivation index (ADI) score *(reference: ADI ≤50)*				0.0188
50< ADI ≤75	1.173	0.955	1.441	
ADI >75	1.380	1.103	1.726	
Age	0.985	0.973	0.998	0.0241
Sex *(reference: Male)*				0.0064
Female	0.408	0.214	0.777	
Race *(reference: White)*				0.2160
Black	1.208	0.962	1.518	
Other	0.593	0.238	1.475	
Unknown	0.739	0.266	2.050	
Body mass index *(reference: 18.5-24.9)*				0.0374
<18.5	0.505	0.277	0.923	
25-29.9	0.799	0.654	0.977	
30-34.9	0.763	0.597	0.974	
≥35	0.818	0.585	1.143	
Smoking status at surgery *(reference: Current)*				0.4803
Never	1.309	0.645	2.655	
Former	1.099	0.919	1.314	
Charlson-Deyo Comorbidity Index score	1.089	1.043	1.138	0.0001
American Society of Anesthesiologists Class	1.452	1.199	1.758	0.0001

Preoperative forced expiratory volume (FEV) in 1 second *(reference: FEV1 ≥80% predicted)*				0.9860
FEV1 50-79% predicted	0.988	0.828	1.178	
FEV1 <50% predicted	1.009	0.710	1.434	
Number of prescription medications in the year before surgery	1.008	0.997	1.020	0.1402
Distance from treatment facility *(reference: 0-10 miles)*				0.0008
11-50 miles	1.046	0.841	1.299	
>50 miles	0.717	0.567	0.907	
Annual hospital case volume	0.997	0.995	0.998	<0.0001
Tumor size *(reference ≤10 mm)*				0.6626
11-20 mm	0.789	0.582	1.068	
21-30 mm	0.851	0.621	1.167	
31-40 mm	0.891	0.630	1.259	
41-50 mm	0.755	0.497	1.145	
Unknown	<0.001	<0.001	>999.999	
Tumor grade *(reference: I)*				0.0622
II	0.864	0.660	1.129	
III	1.081	0.816	1.432	
IV	1.488	0.753	2.940	
Tumor histology *(reference: Adenocarcinoma)*				0.0165
Squamous cell carcinoma	1.051	0.871	1.269	
Other	0.663	0.486	0.905	
Tumor location *(reference: Right upper lobe)*				0.4128
Left lower lobe	0.826	0.630	1.084	
Left upper lobe	0.961	0.781	1.183	
Right lower or middle lobe	0.860	0.682	1.086	
Surgical approach *(reference: Thoracotomy)*				0.5108
Minimally invasive	0.939	0.779	1.132	
Lung resection type *(reference: Lobectomy)*				0.1373
Pneumonectomy	1.440	0.825	2.513	
Segmentectomy	0.727	0.477	1.110	
Wedge resection	0.847	0.672	1.068	
Adequate lymph node sampling *(reference: <three N2 and one N1 nodal stations)*				0.0004
≥three N2 and one N1 nodal stations	1.399	1.162	1.684	
Year of operation	0.962	0.933	0.992	0.0138
Pathologic Upstaging Present *(reference: No)*				0.0027
Yes	1.420	1.130	1.786	
Met All Preoperative Care Quality Measures *(reference: No)*				0.0737
Yes	1.177	0.985	1.406	

Supplemental Table S4. Results from multivariable regression model constructed to evaluate the relationship between area deprivation index and 30-day major postoperative complications.

Parameter	Adjusted Odds Ratio	Lower Limit of 95% Confidence Interval	Upper Limit of 95% Confidence Interval	P-value
Area deprivation index (ADI) score *(reference: ADI ≤50)*				0.2739
50< ADI ≤75	1.056	0.908	1.228	
ADI >75	0.927	0.780	1.101	
Age	0.999	0.990	1.009	0.8923
Sex *(reference: Male)*				0.1017
Female	0.713	0.476	1.069	
Race *(reference: White)*				0.5525
Black	0.910	0.754	1.098	
Other	1.130	0.660	1.934	
Unknown	1.322	0.722	2.421	
Body mass index *(reference: 18.5-24.9)*				0.0008
<18.5	1.513	1.092	2.095	
25-29.9	0.883	0.758	1.028	
30-34.9	0.753	0.624	0.909	
≥35	0.943	0.734	1.211	
Smoking status at surgery *(reference: Current)*				0.3934
Never	0.603	0.288	1.261	
Former	1.009	0.882	1.153	
Charlson-Deyo Comorbidity Index score	1.154	1.117	1.193	<0.0001
American Society of Anesthesiologists Class	1.358	1.174	1.571	<0.0001
Preoperative forced expiratory volume (FEV) in 1 second *(reference: FEV1 ≥80% predicted)*				<0.0001
FEV1 50-79% predicted	1.332	1.165	1.523	
FEV1 <50% predicted	1.579	1.229	2.029	
Number of prescription medications in the year before surgery	1.011	1.002	1.019	0.0139
Distance from treatment facility *(reference: 0-10 miles)*				0.1509
11-50 miles	0.848	0.716	1.004	
>50 miles	0.921	0.774	1.097	
Annual hospital case volume	1.000	0.998	1.001	0.4648
Tumor size *(reference ≤10 mm)*				0.6772
11-20 mm	1.009	0.790	1.288	
21-30 mm	0.930	0.721	1.200	
31-40 mm	1.067	0.811	1.403	
41-50 mm	1.121	0.820	1.534	
Unknown	<0.001	<0.001	>999.999	
Tumor grade *(reference: I)*				0.0047
II	0.955	0.774	1.177	
III	1.216	0.977	1.515	
IV	0.816	0.453	1.469	

Tumor histology *(reference: Adenocarcinoma)*				0.1467
Squamous cell carcinoma	1.148	0.996	1.323	
Other	1.111	0.904	1.366	
Tumor location *(reference: Right upper lobe)*				0.1219
Left lower lobe	0.884	0.724	1.079	
Left upper lobe	0.825	0.703	0.968	
Right lower or middle lobe	0.934	0.788	1.107	
Surgical approach *(reference: Thoracotomy)*				<0.0001
Minimally invasive	0.656	0.568	0.758	
Lung resection type *(reference: Lobectomy)*				<0.0001
Pneumonectomy	1.722	1.134	2.616	
Segmentectomy	0.665	0.487	0.908	
Wedge resection	0.584	0.488	0.700	
Adequate lymph node sampling *(reference: <three N2 and one N1 nodal stations)*				0.1579
≥three N2 and one N1 nodal stations	1.109	0.961	1.280	
Year of operation	0.968	0.946	0.991	0.0060
Pathologic Upstaging Present *(reference: No)*				0.2967
Yes	1.104	0.917	1.330	
Met All Preoperative Care Quality Measures *(reference: No)*				0.2502
Yes	1.083	0.946	1.239	

Supplemental Table S5. Results from multivariable regression model constructed to evaluate the relationship between area deprivation index and 30-day postoperative mortality.

Parameter	Adjusted Odds Ratio	Lower Limit of 95% Confidence Interval	Upper Limit of 95% Confidence Interval	P-value
Area deprivation index (ADI) score *(reference: ADI ≤50)*				0.4447
50< ADI ≤75	0.970	0.662	1.421	
ADI >75	1.221	0.816	1.826	
Age	1.040	1.017	1.064	0.0007
Sex *(reference: Male)*				0.8988
Female	1.069	0.384	2.976	
Race *(reference: White)*				0.5522
Black	1.136	0.734	1.758	
Other	1.269	0.370	4.358	
Unknown	2.233	0.677	7.364	
Body mass index *(reference: 18.5-24.9)*				0.0003
<18.5	2.767	1.529	5.006	
25-29.9	0.735	0.505	1.069	
30-34.9	0.650	0.405	1.041	
≥35	0.804	0.428	1.512	
Smoking status at surgery *(reference: Current)*				0.9194
Never	0.659	0.089	4.877	
Former	0.988	0.714	1.368	
Charlson-Deyo Comorbidity Index score	1.138	1.053	1.229	0.0011

American Society of Anesthesiologists Class	1.717	1.231	2.395	0.0014
Preoperative forced expiratory volume (FEV) in 1 second *(reference: FEV1 ≥80% predicted)*				0.1860
FEV1 50-79% predicted	1.233	0.888	1.711	
FEV1 <50% predicted	1.646	0.923	2.937	
Number of prescription medications in the year before surgery	1.006	0.985	1.027	0.5873
Distance from treatment facility *(reference: 0-10 miles)*				0.6955
11-50 miles	0.888	0.592	1.331	
>50 miles	0.833	0.546	1.270	
Annual hospital case volume	1.001	0.998	1.004	0.3631
Tumor size *(reference ≤10 mm)*				0.5006
11-20 mm	1.743	0.825	3.683	
21-30 mm	1.464	0.679	3.158	
31-40 mm	2.034	0.921	4.488	
41-50 mm	1.512	0.625	3.659	
Unknown	<0.001	<0.001	>999.999	
Tumor grade *(reference: I)*				0.8508
II	1.130	0.647	1.971	
III	1.256	0.705	2.240	
IV	0.999	0.213	4.686	
Tumor histology *(reference: Adenocarcinoma)*				0.1454
Squamous cell carcinoma	1.381	0.982	1.942	
Other	1.003	0.584	1.723	
Tumor location *(reference: Right upper lobe)*				0.2945
Left lower lobe	0.674	0.389	1.170	
Left upper lobe	1.112	0.759	1.631	
Right lower or middle lobe	1.130	0.750	1.703	
Surgical approach *(reference: Thoracotomy)*				0.3785
Minimally invasive	0.851	0.594	1.219	
Lung resection type *(reference: Lobectomy)*				<0.0001
Pneumonectomy	6.851	3.802	12.347	
Segmentectomy	0.203	0.050	0.830	
Wedge resection	0.599	0.384	0.932	
Adequate lymph node sampling *(reference: <three N2 and one N1 nodal stations)*				0.4402
≥three N2 and one N1 nodal stations	1.145	0.811	1.617	
Year of operation	0.940	0.888	0.996	0.0350
Pathologic Upstaging Present *(reference: No)*				0.1563
Yes	1.357	0.890	2.070	
Met All Preoperative Care Quality Measures *(reference: No)*				0.0168
Yes	0.643	0.448	0.924	0.643

Supplemental Table S6. Results from multivariable regression model constructed to evaluate the relationship between area deprivation index and 90-day postoperative mortality.

Parameter	Adjusted Odds Ratio	Lower Limit of 95% Confidence Interval	Upper Limit of 95% Confidence Interval	P-value
Area deprivation index (ADI) score *(reference: ADI ≤50)*				0.6993
50< ADI ≤75	0.943	0.720	1.237	
ADI >75	0.876	0.645	1.190	
Age	1.035	1.018	1.053	<0.0001
Sex *(reference: Male)*				0.3601
Female	0.654	0.264	1.623	
Race *(reference: White)*				0.4835
Black	0.855	0.605	1.209	
Other	1.141	0.435	2.993	
Unknown	1.792	0.702	4.578	
Body mass index *(reference: 18.5-24.9)*				<0.0001
<18.5	2.800	1.764	4.444	
25-29.9	0.711	0.539	0.937	
30-34.9	0.598	0.419	0.851	
≥35	1.013	0.660	1.556	
Smoking status at surgery *(reference: Current)*				0.3579
Never	0.390	0.053	2.850	
Former	1.134	0.893	1.440	
Charlson-Deyo Comorbidity Index score	1.151	1.087	1.219	<0.0001
American Society of Anesthesiologists Class	1.391	1.080	1.790	0.0105
Preoperative forced expiratory volume (FEV) in 1 second *(reference: FEV1 ≥80% predicted)*				0.0165
FEV1 50-79% predicted	1.286	1.009	1.641	
FEV1 <50% predicted	1.746	1.150	2.652	
Number of prescription medications in the year before surgery	1.006	0.991	1.022	0.4355
Distance from treatment facility *(reference: 0-10 miles)*				0.6626
11-50 miles	0.870	0.644	1.176	
>50 miles	0.907	0.664	1.240	
Annual hospital case volume	1.000	0.997	1.002	0.6970
Tumor size *(reference ≤10 mm)*				0.0179
11-20 mm	1.919	1.065	3.455	
21-30 mm	1.920	1.055	3.493	
31-40 mm	2.705	1.461	5.005	
41-50 mm	2.709	1.398	5.250	
Unknown	<0.001	<0.001	>999.999	
Tumor grade *(reference: I)*				0.6622
II	1.234	0.810	1.880	
III	1.319	0.852	2.041	
IV	1.375	0.525	3.604	
Tumor histology *(reference: Adenocarcinoma)*				0.1074
Squamous cell carcinoma	1.309	1.016	1.685	

Other	1.217	0.836	1.771	
Tumor location *(reference: Right upper lobe)*				0.0301
Left lower lobe	0.548	0.367	0.819	
Left upper lobe	0.837	0.633	1.107	
Right lower or middle lobe	0.812	0.599	1.101	
Surgical approach *(reference: Thoracotomy)*				0.0119
Minimally invasive	0.710	0.543	0.927	
Lung resection type *(reference: Lobectomy)*				<0.0001
Pneumonectomy	4.207	2.499	7.080	
Segmentectomy	0.499	0.252	0.987	
Wedge resection	0.739	0.540	1.012	
Adequate lymph node sampling *(reference: <three N2 and one N1 nodal stations)*				0.9004
≥three N2 and one N1 nodal stations	1.017	0.782	1.322	
Year of operation	0.939	0.900	0.979	0.0033
Pathologic Upstaging Present *(reference: No)*				0.0278
Yes	1.419	1.039	1.938	
Met All Preoperative Care Quality Measures *(reference: No)*				0.0110
Yes	0.716	0.553	0.926	

Supplemental Table S7. Results from multivariable regression model constructed to evaluate the relationship between area deprivation index and overall survival.

Parameter	Adjusted Hazard Ratio	Lower Limit of 95% Confidence Interval	Upper Limit of 95% Confidence Interval	P-value
Area deprivation index (ADI) score *(reference: ADI ≤50)*				0.6432
50< ADI ≤75	0.968	0.904	1.036	
ADI >75	0.984	0.911	1.062	
Age	1.024	1.019	1.028	<0.0001
Sex *(reference: Male)*				<0.0001
Female	0.657	0.547	0.788	
Race *(reference: White)*				0.0002
Black	0.826	0.758	0.901	
Other	0.991	0.769	1.277	
Unknown	1.079	0.798	1.458	
Body mass index *(reference: 18.5-24.9)*				<0.0001
<18.5	1.443	1.241	1.677	
25-29.9	0.773	0.722	0.827	
30-34.9	0.714	0.657	0.777	
≥35	0.760	0.675	0.856	
Smoking status at surgery *(reference: Current)*				0.0014
Never	0.618	0.455	0.841	
Former	0.934	0.879	0.993	
Charlson-Deyo Comorbidity Index score	1.075	1.059	1.092	<0.0001
American Society of Anesthesiologists Class	1.263	1.182	1.350	<0.0001

Preoperative forced expiratory volume (FEV) in 1 second *(reference: FEV1 ≥80% predicted)*				<0.0001
FEV1 50-79% predicted	1.189	1.119	1.263	
FEV1 <50% predicted	1.333	1.194	1.488	
Number of prescription medications in the year before surgery	1.016	1.012	1.020	<0.0001
Distance from treatment facility *(reference: 0-10 miles)*				0.0980
11-50 miles	0.992	0.919	1.071	
>50 miles	0.930	0.859	1.007	
Annual hospital case volume	1.000	1.000	1.001	0.8142
Tumor size *(reference ≤10 mm)*				<0.0001
11-20 mm	0.980	0.879	1.092	
21-30 mm	1.048	0.937	1.173	
31-40 mm	1.247	1.104	1.410	
41-50 mm	1.270	1.100	1.466	
Unknown	0.000	0.000	3.187E39	
Tumor grade *(reference: I)*				<0.0001
II	1.240	1.124	1.369	
III	1.369	1.234	1.519	
IV	1.331	1.042	1.700	
Tumor histology *(reference: Adenocarcinoma)*				0.0118
Squamous cell carcinoma	1.060	0.994	1.130	
Other	1.142	1.041	1.252	
Tumor location *(reference: Right upper lobe)*				0.0453
Left lower lobe	0.996	0.910	1.090	
Left upper lobe	0.980	0.911	1.053	
Right lower or middle lobe	1.093	1.012	1.180	
Surgical approach *(reference: Thoracotomy)*				0.1549
Minimally invasive	0.955	0.896	1.018	
Lung resection type *(reference: Lobectomy)*				<0.0001
Pneumonectomy	1.264	1.021	1.565	
Segmentectomy	1.054	0.924	1.203	
Wedge resection	1.277	1.188	1.373	
Adequate lymph node sampling *(reference: <three N2 and one N1 nodal stations)*				0.2709
≥three N2 and one N1 nodal stations	0.963	0.901	1.030	
Year of operation	0.958	0.947	0.969	<0.0001
Pathologic Upstaging Present *(reference: No)*				<0.0001
Yes	1.698	1.565	1.843	
Met All Preoperative Care Quality Measures *(reference: No)*				0.0080
Yes	0.919	0.864	0.978	

Supplemental Table S8. Results from multivariable regression model constructed to evaluate the relationship between area deprivation index and cumulative incidence of cancer recurrence.

Parameter	Adjusted Hazard Ratio	Lower Limit of 95% Confidence Interval	Upper Limit of 95% Confidence Interval	P-value
Area deprivation index (ADI) score *(reference: ADI ≤50)*				0.5476
50< ADI ≤75	1.060	0.953	1.180	
ADI >75	1.047	0.930	1.179	
Age	0.988	0.982	0.995	0.0008
Sex *(reference: Male)*				0.2249
Female	0.859	0.672	1.098	
Race *(reference: White)*				0.5561
Black	0.992	0.874	1.127	
Other	0.785	0.522	1.181	
Unknown	0.787	0.458	1.352	
Body mass index *(reference: 18.5-24.9)*				0.8058
<18.5	1.141	0.897	1.452	
25-29.9	1.003	0.900	1.117	
30-34.9	1.012	0.887	1.154	
≥35	0.952	0.792	1.145	
Smoking status at surgery *(reference: Current)*				0.5086
Never	1.245	0.860	1.801	
Former	1.005	0.913	1.105	
Charlson-Deyo Comorbidity Index score	0.999	0.974	1.024	0.9169
American Society of Anesthesiologists Class	1.081	0.970	1.205	0.1594
Preoperative forced expiratory volume (FEV) in 1 second *(reference: FEV1 ≥80% predicted)*				0.4299
FEV1 50-79% predicted	1.055	0.961	1.159	
FEV1 <50% predicted	0.971	0.805	1.171	
Number of prescription medications in the year before surgery	1.000	0.994	1.006	0.9380
Distance from treatment facility *(reference: 0-10 miles)*				<0.0001
11-50 miles	0.908	0.809	1.020	
>50 miles	0.733	0.648	0.829	
Annual hospital case volume	1.001	1.000	1.001	0.1345
Tumor size *(reference ≤10 mm)*				<0.0001
11-20 mm	1.134	0.952	1.351	
21-30 mm	1.376	1.149	1.647	
31-40 mm	1.503	1.235	1.829	
41-50 mm	1.483	1.174	1.872	
Unknown	10.316	7.706	13.809	
Tumor grade *(reference: I)*				0.0003
II	1.373	1.176	1.604	
III	1.426	1.210	1.682	
IV	1.408	0.938	2.114	
Tumor histology *(reference: Adenocarcinoma)*				0.0029

Squamous cell carcinoma	0.845	0.763	0.937	
Other	1.024	0.886	1.184	
Tumor location *(reference: Right upper lobe)*				0.4621
Left lower lobe	1.056	0.917	1.217	
Left upper lobe	0.995	0.888	1.114	
Right lower or middle lobe	1.086	0.963	1.225	
Surgical approach *(reference: Thoracotomy)*				0.9120
Minimally invasive	0.994	0.901	1.097	
Lung resection type *(reference: Lobectomy)*				<0.0001
Pneumonectomy	0.756	0.511	1.119	
Segmentectomy	1.307	1.080	1.584	
Wedge resection	1.355	1.209	1.517	
Adequate lymph node sampling *(reference: <three N2 and one N1 nodal stations)*				0.0962
≥three N2 and one N1 nodal stations	0.914	0.823	1.016	
Year of operation	0.980	0.964	0.996	0.0146
Pathologic Upstaging Present *(reference: No)*				<0.0001
Yes	1.510	1.334	1.708	
Met All Preoperative Care Quality Measures *(reference: No)*				0.8266
Yes	1.011	0.919	1.112	
